# Functionally aberrant electrophysiological cortical connectivities in first episode medication-naive schizophrenics from three psychiatry centers

**DOI:** 10.3389/fnhum.2014.00635

**Published:** 2014-08-20

**Authors:** Dietrich Lehmann, Pascal L. Faber, Roberto D. Pascual-Marqui, Patricia Milz, Werner M. Herrmann, Martha Koukkou, Naomi Saito, Georg Winterer, Kieko Kochi

**Affiliations:** ^1^Department of Psychiatry, Psychotherapy and Psychosomatics, The KEY Institute for Brain-Mind Research, University Hospital for PsychiatryZurich, Switzerland; ^2^Laboratory of Clinical Psychophysiology, Department of Psychiatry, University Hospital Benjamin Franklin, Free University of BerlinBerlin, Germany; ^3^Saito Mental ClinicOsaka, Japan; ^4^Experimental and Clinical Research Center, Charité – University Medicine BerlinBerlin, Germany

**Keywords:** functional connectivity, functional dissociation, EEG, LORETA, schizophrenia subtypes, cortical source modeling, electrical source imaging

## Abstract

Functional dissociation between brain processes is widely hypothesized to account for aberrations of thought and emotions in schizophrenic patients. The typically small groups of analyzed schizophrenic patients yielded different neurophysiological findings, probably because small patient groups are likely to comprise different schizophrenia subtypes. We analyzed multichannel eyes-closed resting EEG from three small groups of acutely ill, first episode productive schizophrenic patients before start of medication (from three centers: Bern *N* = 9; Osaka *N* = 9; Berlin *N* = 12) and their controls. Low resolution brain electromagnetic tomography (LORETA) was used to compute intracortical source model-based lagged functional connectivity not biased by volume conduction effects between 19 cortical regions of interest (ROIs). The connectivities were compared between controls and patients of each group. Conjunction analysis determined six aberrant cortical functional connectivities that were the same in the three patient groups. Four of these six concerned the facilitating EEG alpha-1 frequency activity; they were decreased in the patients. Another two of these six connectivities concerned the inhibiting EEG delta frequency activity; they were increased in the patients. The principal orientation of the six aberrant cortical functional connectivities was sagittal; five of them involved both hemispheres. In sum, activity in the posterior brain areas of preprocessing functions and the anterior brain areas of evaluation and behavior control functions were compromised by either decreased coupled activation or increased coupled inhibition, common across schizophrenia subtypes in the three patient groups. These results of the analyzed three independent groups of schizophrenics support the concept of functional dissociation.

## Introduction

The brain mechanisms that implement the symptomatology of schizophrenia early on elicited the concept of dissociation of mental functions (Janet, 1889) or of splitting of the psychic functions (Bleuler, 1911/1950) as evident in psychological aberrations, for example in the patients' “double bookkeeping” (Bleuler, 1951), their distorted sense of self (Park and Nasrallah, 2014) and their disturbed attention management (Dichter et al., 2010). In terms of brain mechanisms, the condition was conceptualized as disconnection between the brain's neural networks (Beaumont and Dimond, 1973; Friston, 1996, 1998; Stephan et al., 2009).

This concept of disconnection and thus, reduced cooperation (Jalili et al., 2007) or coordination (Phillips et al., 2010) between psychological brain functions that are physiologically implemented in the activity of spatially distributed neuronal networks (Mesulam, 1990; Tononi et al., 1998) implies an increase of independent brain processes. Therefore, measures of dimensional complexity of the brain's activity should show increased values compared to controls. However, reported EEG or MEG results varied, depending on medication effects, symptomatology, and age as reported in detail in a recent review (Fernandez et al., 2013): During no-task resting, increased complexity was found in first-episode, acutely ill, productive patients before begin of medication (e.g., Koukkou et al., 1993, 1995; Irisawa et al., 2006; Li et al., 2008; Raghavendra et al., 2009; Takahashi et al., 2010) while decreased complexity was found in chronic, older and medicated patients (e.g., Jeong et al., 1998; Kim et al., 2000; Na et al., 2002; Jin et al., 2003; Raghavendra et al., 2009; Takahashi et al., 2010). Thus, medication effects, symptomatology, and age tend to affect the results in opposing directions, which might explain why the dissociation concept does not fit all groups of schizophrenias. The increase of independent brain processes in medication-naïve first episode schizophrenics also agrees with their shortened duration of temporal EEG microstates (Koukkou et al., 1994; Koenig et al., 1999; Lehmann et al., 2005).

Networks are established by functional connectivities between active brain regions. The neurophysiology of mental states is described by the brain's functional connectivities (e.g., Walter, 1963; Walter et al., 1967; Stam, 2000; Mizuhara et al., 2005; Singer, 2009; White et al., 2009). With increasing number of independent brain processes, decreases of EEG coherence are to be expected. Several studies on scalp EEG connectivity in schizophrenia indeed reported decreased EEG coherence (e.g., Flor-Henry and Koles, 1984; Merrin and Floyd, 1992; Tauscher et al., 1998; Winterer et al., 2001), but others reported increases (e.g., Ford et al., 1986; Nagase et al., 1992; Mann et al., 1997; Wada et al., 1998) or increases as well as decreases depending on patients' subtype or medication (Knott et al., 2002; Medkour et al., 2010). Some coherence studies analyzed no-task resting, others task conditions, contributing to the differences in results. As in the EEG complexity studies, medication effects, symptomatology, and age affected the results; coherence tended to decrease in medication-naive, younger, first episode patients.

Studies on first episode schizophrenic patients before medication typically comprise only very few patients. The lower the number of patients, the higher the probability that the different subtypes of schizophrenia are not equally present. Bleuler's book (Bleuler, 1911/1950) on “the group of schizophrenias” in its title already indicated that schizophrenia is a heterogeneous disorder. Patients diagnosed with schizophrenia may present with very different symptoms and accordingly would show different neurophysiological aberrations from healthy controls. Therefore, EEG, fMRI, or PET results may strongly differ between small groups of schizophrenics (Stephan, 2013). But, since all patients are classified as schizophrenics, one would nevertheless expect some communality among the brain mechanisms that subserve functional connectivity between networks and, when decreased, result in functional dissociation.

Computation of coherence between scalp EEG data has met with criticism concerning the methodology. EEG waveforms recorded from electrodes on the scalp depend on the chosen reference (there is no physical proof for a location of zero potential (Geselowitz, 1998). Therefore, coherence between scalp EEG waveforms is ambiguous. Secondly, coherence between scalp EEG waveforms will not reveal true functional connectivity between brain regions because electrical neuronal sources do not necessarily project radially to the scalp: The classical case is the maximal evoked potential P-100 on the scalp contralateral to the visually stimulated hemisphere (Barret et al., 1976; Shagass et al., 1976). Computing EEG coherence between intracerebral generator model sources was suggested to solve these two problems (Ruchkin, 2005). A third issue is that the computation of functional connectivity should avoid effects of volume conduction that erroneously increase coherence; omitting zero phase angle coherence solves this problem (Nolte et al., 2004). Following these three recommendations, for the computation of functional connectivity we used “lagged” coherence (omitting zero phase angle) based on intracortical source models (Pascual-Marqui, 2007a,b; Pascual-Marqui et al., 2011).

Expanding results on first episode schizophrenic patients before medication beyond the many studies with small samples of heterogeneous subtypes, we investigated functional connectivity in three independent groups of acutely ill, medication-naïve, first episode schizophrenic patients comprising different diagnostic subtypes and compared them with healthy controls. We determined the functional connectivities that significantly differed between patients and controls in each group; their co-occurrence across groups was established using conjunction analysis. In order to avoid the ambiguities of source localization, reference-dependence and volume conduction of earlier functional connectivity studies, we computed intracortical lagged coherence using eLORETA.

Based on the disconnection concept of schizophrenia, we hypothesized that across the three diagnostically heterogeneous patient groups, we would find a core set of decreased cortical functional EEG connectivities, identical in our three groups.

## Methods

Data of acutely ill, medication-naïve, first episode schizophrenic patients and their controls were available from university hospitals of psychiatry in Bern (Switzerland), Osaka (Japan), and Berlin (Germany), comprising 9, 9, and 12 patients, respectively, and their 36, 9, and 12 controls. The demographics in Table 1 include the diagnostic subtypes of the patients. All three control groups were recruited among the respective hospital employees, their relatives and students.

**Table 1:** **Demographics**.

**Center**	**Bern**	**Osaka**	**Berlin**
**PATIENTS**			
Women	6	4	2
Men	3	5	10
Mean age (years)	24.8	20.7	25.2
SD (years)	6.7	5.4	4.7
Range (years)	17–38	17–32	18–32
**Diagnostic Subtype**			
Hebephrenic	–	1	–
Catatonic	–	1	–
Paranoid	5	1	12
Schizophreniform	4	–	–
Undifferentiated	–	6	–
Total *N*	9	9	12
**CONTROLS**			
Women	19	4	12
Men	17	5	10
Total *N*	36	9	12
Mean age (years)	28	21.8	25.3
*SD* (years)	5.6	2.4	4.6
Range (years)	17–38	17–32	18–32

Multichannel EEG was recorded against linked earlobes (Bern and Berlin) or against Cz (Osaka). Nineteen electrodes (Fp1/2, F3/4, C3/4, P3/4, O1/2, F7/8, T3/4, T5/6, Fz, Cz, and Pz) were used in Bern and Osaka, 21 electrodes (Fpz and Oz in addition) in Berlin, all placed according to the International 10/20 system. In Bern and Osaka, data were recorded using a BioLogic Brain Atlas and digitized at 128 samples/s, using a bandpass of 1–30 Hz in Bern and of 0.3–30 Hz in Osaka. In Berlin, data were recorded using a Walter Graphtek system and digitized at 166.6 samples/s and bandpassed at 0.3–70 Hz in Berlin.

During recording, participants were comfortably seated in a sound-shielded chamber; they were recorded during no-task resting with closed eyes. The eyes closed EEG was recorded for 4 min in Bern and Berlin and for 3 min in Osaka. In all three centers the first 20 artifact-free 2-s epochs (if available) were used for analysis.

The three datasets had been used earlier for other analyses: the Bern data for EEG microstate analysis (Koenig et al., 1999), for EEG source localization (Pascual-Marqui et al., 1999) and for EEG power spectra (Koukkou et al., 2000), the Osaka and Berlin data for EEG microstate analysis (Lehmann et al., 2005), and the Osaka data for EEG dimensional complexity (Saito et al., 1998).

### EEG pre-processing

All data were screened on a computer display where eye, muscle and technical artifacts were marked for exclusion. The artifact-free data were parsed into analysis epochs of 2 s. On average per patient, there were 18.2 (*SD* = 3.3), 20.0 (*SD* = 0), and 16.1 (*SD* = 4.7) artifact-free 2-s analysis epochs for Bern, Osaka and Berlin, respectively; the corresponding figures for the controls were 20.0 (*SD* = 0), 20.0 (*SD* = 0), and 18.0 (*SD* = 2.9).

The EEG recording characteristics in all three centers covered the frequency bands of delta through beta-2, which are later used in the frequency domain connectivity analyses.

### eLORETA analysis

The pre-processed scalp EEG data were computed into the time varying electric neuronal activity of the cortical sources applying low resolution brain electromagnetic tomography (LORETA, Pascual-Marqui et al., 1994) in order to avoid the ambiguity of source localization and the reference-dependence that is inherent in scalp EEG waveforms. We used the software “exact low-resolution brain electromagnetic tomography” (eLORETA version 20081104, Pascual-Marqui, 2007a; Pascual-Marqui et al., 2011) (free academic software available at <http://www.uzh.ch/keyinst/loreta.htm>).

eLORETA results at each analysis time point consist of current density at each of 6239 cortical voxels (5 mm spatial resolution) in Montreal Neurological Institute (MNI) space (Evans et al., 1993). Reported Brodmann areas (BAs) use MNI space corrected to Talairach space (Brett et al., 2002).

For each analysis epoch, current density for each voxel was computed for the first six of the eight independent EEG frequency bands established by factor analysis (Kubicki et al., 1979; Niedermeyer and Lopes Da Silva, 2005): delta (1.5–6 Hz), theta (6.5–8 Hz), alpha-1 (8.5–10 Hz), alpha-2 (10.5–12 Hz), beta-1 (12.5–18 Hz), and beta-2 (18.5–21 Hz) that were covered by the band-pass of the three datasets. Results were averaged across all analysis epochs for each patient and frequency band. Technical details on the methods for computing the frequency domain cross-spectral matrices of cortical electric neuronal activity can be found in Frei et al. (2001).

### Intracortical functional connectivity computed as “lagged” coherence

The cortical areas under the 19 electrodes Fp1/2, F7/8, F3/4, Fz, C3/4, Cz, T3/T4, T5/6, P3/4, Pz, O1/2 of the “10/20 System” (Jasper, 1958) were used for defining 19 regions of interest (ROIs) in order to make the coherence computations manageable. These cortical areas are well-documented in other low resolution tomographies such as NIRS (Jurcak et al., 2007). The eLORETA option “all nearest voxels” assigned each of the 6239 voxels to one of the 19 ROIs. For each ROI, that BA was determined to which most of the ROI's voxels belonged.

Current density values of all voxels within each given ROI were averaged. Of the intracortical current density time series of all 171 (= 19 ^*^ 18/2) pairs of ROIs for each frequency band and participant, intracortical “lagged” coherences omitting zero phase angle values (in order to remove volume conduction effects) were computed (Pascual-Marqui, 2007b; Pascual-Marqui et al., 2011).

The lagged coherence definition is based on the formulation of the two main components of a connection: instantaneous and lagged. The lagged component can only be mediated by physiological time delays. Based on a proper statistical formulation of this model, any instantaneous contribution to the connectivity is appropriately accounted for and eliminated, leaving connectivity that solely due to physiology (for any non-zero, measurable time delay), not confounded by low resolution and volume conduction effects. The technical details of the derivation can be found in Pascual-Marqui (2007b) and Pascual-Marqui et al. (2011).

Using unpaired Welch *t*-test statistics on the connectivity values, the eLORETA intracortical lagged connectivities were compared between controls and patients of each dataset in 171 tests between patients and controls for each of the six independent EEG frequency bands. The next sub-section on conjunction analysis clarifies the non-corrected thresholds used for statistical inference.

### Conjunction analysis of the results of the three datasets

Conjunction analysis (Friston et al., 1999; Nichols et al., 2005) was used to identify those significantly different connectivities between patients and controls that were common in the three datasets. In a conjunction analysis of three independent tests, significance for a given voxel of a given frequency band is reached if the maximum one-tailed *p*-value corrected for multiple testing is smaller than the cubic root of 0.05, i.e., the three voxels must satisfy *p* < 0.368 (Friston et al., 1999). One-tailed *p*-values provide a meaningful conjunction in which the participating three statistics (*t*-values) show the same sign (i.e., they have the same effect). We report results for non-corrected conjunction *p*-values smaller than *p* = 0.001 and *p* = 0.01 (corresponding to single maximum *p*-values of 10^−5^ and 10^−4^, respectively). Although not corrected for multiple testing, these very extreme thresholds are deemed acceptable for reporting results of statistical significance.

*Post-hoc* unpaired *t*-tests were used to compare the coordinates of the ROIs involved in connectivities significantly different between patients and controls between frequency bands on the left-right and anterior-posterior axis.

### Common spatial tendency across connectivity results

The mean three-dimensional spatial location and orientation of all (in the conjunction analysis) qualifying functional connectivities of each frequency band was computed using “principal functional connectivity” (Lehmann et al., 2012) that represents the three-dimensional least error compromise of all participating connectivities. The computation of the principal functional connectivities is based on the ROI coordinates in MNI-space (gravity center of all constituting voxels) and their node coordinates are therefore also in the MNI-space.

## Results

### Results at non-corrected conjunction *p* < 0.001

**Figure 1:**
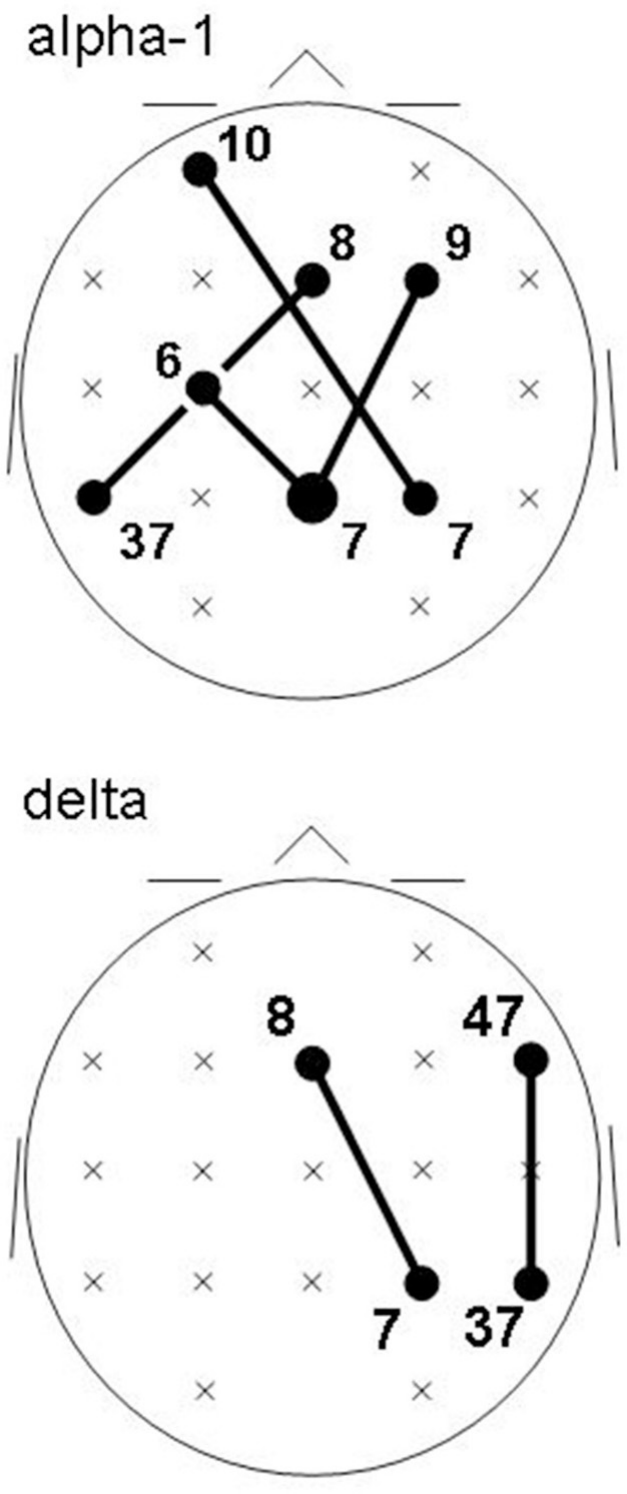
**Common intracortical connectivities differing between patients and controls in all three groups (lines) at non-corrected conjunction *p* < 0.001**. Connectivities of the EEG alpha-1 frequency band (top) are decreased in patients; connectivities in the delta frequency band (bottom) are increased in patients. Head seen from above; semi schematic array of the 19 ROIs. Bubble sizes indicate how many connectivities anchored at each ROI (here, 1 or 2); crosses indicate ROIs where no connectivity anchored. Numbers indicate the Brodmann areas to which most of the voxels of a given ROI were assigned.

**Figure 2:**
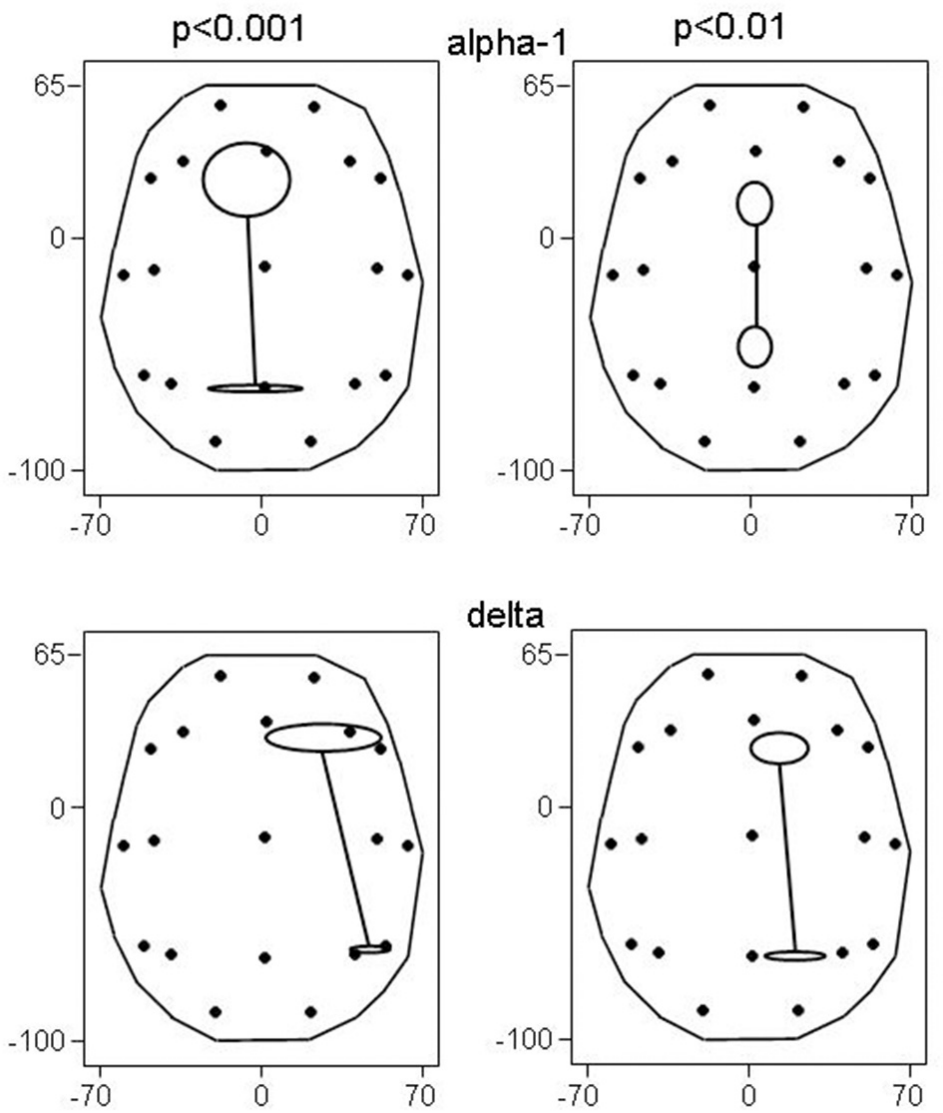
**Principal functional connectivities (and their SE) at non-corrected conjunction *p* < 0.001 and *p* < 0.01**. At *p* < 0.001, 4 connectivities in the alpha-1 band, and 2 connectivities in the delta band qualified; at *p* < 0.01, there were 26 and 9 connectivities, respectively. Head seen above, nose up; contours delineate the eLORETA result space (millimeters are indicated); dots show the 19 ROIs in glass brain view.

Recall that conjunction analysis (Friston et al., 1999; Nichols et al., 2005) was used to identify those significantly different connectivities between patients and controls that were common in the three datasets. This analysis showed that at non-corrected conjunction *p* < 0.001 across the functional connectivities of the three datasets, 4 connectivities in the alpha-1 band were decreased in all three datasets, while 2 connectivities in the delta band were increased in all three datasets. Table 2 shows the connectivity values of the 6 qualifying connectivities from the three centers. These connectivities are illustrated in Figure 1 which also indicates the BAs to which most of the voxels of a given ROI were assigned. Table 3 lists these BAs with the number of voxels concerned and their percentage of all voxels of the respective ROI. The connectivities were: anterior left—posterior right and midline, anterior right—posterior midline, anterior midline—posterior left and right, and anterior right—posterior right. Thus, five involved both hemispheres. Yet, the orientation of the principal functional connectivity of the connectivities for both alpha-1 and delta was anterior-posterior (Figure 2).

**Table 2:** **The connectivity values of the 6 qualifying connectivities from the three centers**.

		**Bern**				**Osaka**				**Berlin**			
		**Controls**		**Patients**		**Controls**		**Patients**		**Controls**		**Patients**	
**Hz band**	**ROIs**	**mean**	***SD***	**mean**	***SD***	**mean**	***SD***	**mean**	***SD***	**mean**	***SD***	**mean**	***SD***
**Alpha-1**	1 and 16	0.276	0.197	0.203	0.110	0.344	0.137	0.205	0.143	0.340	0.191	0.238	0.134
	5 and 13	0.384	0.293	0.277	0.196	0.385	0.127	0.246	0.140	0.434	0.289	0.255	0.186
	6 and 15	0.319	0.253	0.237	0.112	0.520	0.305	0.186	0.105	0.500	0.153	0.304	0.211
	9 and 15	0.488	0.359	0.304	0.312	0.514	0.450	0.285	0.199	0.621	0.391	0.423	0.183
**Delta**	5 and 16	0.075	0.041	0.096	0.039	0.075	0.049	0.143	0.140	0.079	0.036	0.119	0.058
	7 and 17	0.081	0.038	0.103	0.031	0.059	0.025	0.084	0.037	0.081	0.039	0.125	0.067

**Figure 3:**
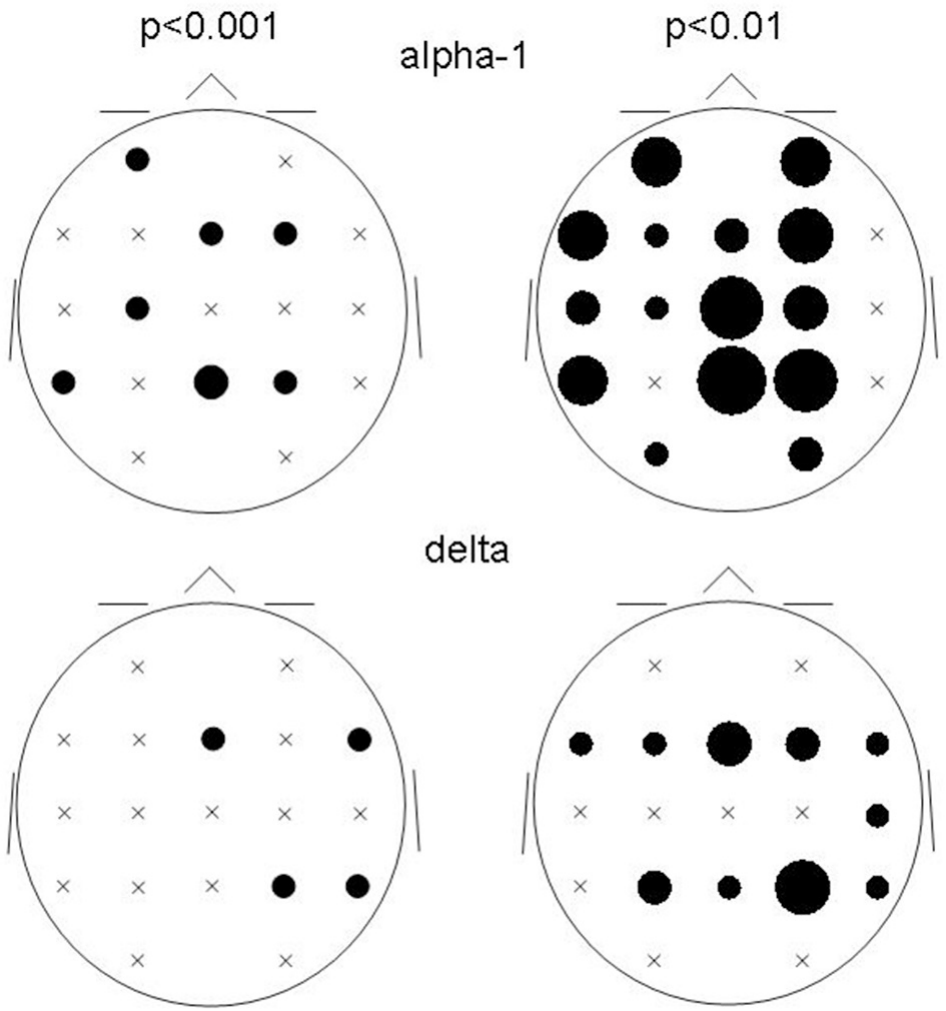
**Bubble sizes indicate how many connectivities anchored at each ROI in the comparison between patients and controls**. Head seen from above. Semi schematic array of the 19 ROIs; crosses indicate ROIs where no connectivity anchored. Results for the alpha-1 and delta frequency bands are shown for the two non-corrected conjunction *p*-values of 0.01 and 0.001.

*The connectivities are defined by the involved ROIs. The 19 ROIs (crosses in Figures 1, 3) are numbered first left to right, then anterior to posterior*.

**Table 3:** **ROIs and the BAs contributing most voxels to the ROIs**.

**ROI**	**BA**	**Number of voxels in BA**	**% of ROI voxels in BA**
1	10	85	45.2
5	8	78	25.2
6	9	79	33.2
7	47	109	28.8
9	6	138	30.7
13	37	90	28.6
15	7	231	65.3
16	7	88	34.5
17	37	82	26.4

*Listed are ROIs with significant connectivities in the delta or alpha-1 band, the number of contributing voxels of the BA and the percentage of these voxels in the ROI*.

The involved seven ROIs in the alpha-1 band were significantly more to the left (*p* = 0.044) than the involved four ROIs of the delta band. These delta band connectivities were predominantly in the right hemisphere. There was no significant difference in the anterior-posterior direction between alpha-1 and delta band ROIs.

### Results at non-corrected conjunction *p* < 0.01

In order to check whether the obtained results reflect the general tendency of the data, we examined the results at lowered statistical thresholding, at non-corrected conjunction *p* < 0.01. At this level, 51 cortical functional connectivities qualified. And indeed, the results supported the above findings at *p* < 0.001, showing a corresponding profile of occurrences (Table 4): 26 of all 38 decreases occurred in alpha-1, and 9 of all 13 increases occurred in delta.

**Table 4:** **Number of connectivities that showed decreases or increases in patients compared to controls in the three datasets as determined by conjunction analysis at the two non-corrected conjunction *p*-levels of 0.001 and 0.01**.

	**Decreased**		**Increased**	
	*****p*** < **0.001****	*****p*** < **0.01****	*****p*** < **0.001****	*****p*** < **0.01****
**FREQUENCY Hz BAND**				
Delta	–	–	2	9
Theta	–	6	–	–
Alpha-1	4	26	–	–
Alpha-2	–	–	–	1
Beta-1	–	3	–	1
Beta-2	–	3	–	2
Total	4	38	2	13

As in the above results at *p* < 0.001, the alpha-1 band ROIs tended to be more to the left than the delta band ROIs (*p* < 0.086); again there was no difference in the anterior-posterior direction, and again the delta band connectivities were predominantly in the right hemisphere.

Also, the principal functional connectivity of the connectivities in both the alpha-1 and delta frequency band was oriented in the anterior-posterior direction (Figure 2) as in the results at *p* < 0.001. It is noteworthy that not all bands had an anterior-posterior-oriented principal functional connectivity: At *p* < 0.01, the 3 cases of decreased connectivity in the beta-1 band showed a left-right-oriented principal functional connectivity.

The spatial distribution of the involved ROIs for the results obtained at *p* < 0.001 and *p* < 0.01 were quite comparable for the delta and alpha-1 bands (Figure 3): While alpha-1 showed no cases at the rightmost ROI column for both *p*-levels, delta did.

## Discussion

In line with our hypothesis, the three independent small groups of acutely ill, first episode schizophrenic patients (before medication) comprising heterogeneous subtypes showed some identical decreased cortical functional connectivities. The decreases were found in the alpha-1 EEG frequency band. Interestingly we also observed some identical increased cortical functional connectivities. The increases concerned the delta band. The results suggest that there is a common fundamental neurophysiological mal-functioning across heterogeneous, acutely ill, first episode, medication-naive schizophrenics.

Increased alpha activity has been associated with internally directed attention (Cooper et al., 2003), a relaxed state of alert wakefulness (Klimesch, 1999; Müller et al., 1999; Irisawa et al., 2006), active memory processes (Palva and Palva, 2007) and sensory functions (Schurmann et al., 1997). The alpha rhythm also reflects the anticipatory processing of events (Karakas, 1997; Klimesch, 1999). Alpha activity can be regarded as a facilitatory activity: it facilitates learning (Sigala et al., 2014) and normal internal mental processes (Knyazev et al., 2011). We note that for the upper alpha band, power increases were also linked to active inhibition in favor of neighboring brain areas (Klimesch, 1996; Pfurtscheller, 2003; Klimesch et al., 2007). However, our results concerned only the alpha-1 band.

The present results show a clear decrease in connectivities between brain areas in the alpha-1 EEG frequency band, indicating a disturbed cooperation between these areas in patients compared to controls.

Delta assumedly inhibits brain functions (Makeig and Jung, 1995; Niedermeyer and Lopes Da Silva, 2005; O'gorman et al., 2013). In the resting eyes closed awake condition, an excess of delta activity and or connectivity corresponds to dysfunction, i.e., abnormal function. If one cortical area transmits or shares a stronger than normal amount of delta oscillations with another cortical area, then they are transmitting and sharing activity that by its very nature inhibits normal functioning.

The decreased coupled facilitation was more on the left, the increased coupled inhibition more on the right. In both cases, the dominant direction of the aberrant interaction computed as principal functional connectivity was anterior-posterior. All six cases concerned connectivities between anterior brain areas of evaluation and behavior control functions (left BA6, bilateral BA8, right BA9, left BA10, right BA47) and posterior brain areas of preprocessing functions (right BA7, bilateral BA7, left BA37, right BA37). The principal functional connectivity of the six aberrant cortical functional connectivities was saggitaly oriented, indicating a degradation of the functional connectivity between frontal and posterior regions. This would concern the fronto-parietal network of attention (Markett et al., 2013) whose function is compromised in schizophrenia (Dichter et al., 2010; Smucny et al., 2013), apparently at very early stages of information processing (Smucny et al., 2013). The observed (delta frequency) right-hemispheric inhibition of this connectivity was also noted in a MEG study of first episode schizophrenics (Roiser et al., 2013). Five of the six aberrant connectivities involved both hemispheres, indicating a degradation of the functional connectivity between hemispheres in the patients. fMRI studies which examined inter-hemispheric functional connectivity in schizophrenics and their siblings also had reported reduced connectivity albeit concerning very slow frequencies below 0.1 Hz (Hoptman et al., 2012; Guo et al., 2014a,b). The size of the corpus callosum reportedly is reduced in schizophrenia, in particular in first episode patients (Arnone et al., 2008).

The results above at non-corrected conjunction *p* < 0.001 were supported by the results at *p* < 0.01 which showed that the increased delta and decreased alpha-1 frequency band connectivities contributed 68% of the qualifying connectivities (Table 2), again of anterior-posterior-oriented principal functional connectivity (Figure 2).

It is important to clarify that the reason for this two-step analysis strategy using two different thresholds is partly due to the technical problem of determining the exact corrected thresholds. Unfortunately, these ideal threshold values are not available. Nor would it be correct to use a Bonferroni-type threshold, because of the highly correlated structure of all variables estimated from eLORETA (due to its low spatial resolution). For this reason, the results were examined first at an extremely high non-corrected single test threshold (*p* < 10^−5^), to ensure a low declaration of false positives. However, since the exact threshold is unknown, these results might be overly conservative. Therefore, a second examination at a slightly lower threshold (single test uncorrected *p* < 10^−4^) was performed, which in this case simply validated the first more conservative results.

The relation of the compromised connectivities to schizophrenic symptomatology must remain speculative since many functions that are relevant in schizophrenic symptomatology have been ascribed to several BAs in fMRI and PET studies on normal participants. For the six compromised connectivities that were commonly disturbed in our three patient groups, the literature about functions of the involved BAs offers the following possibilities (in BA sequence: anterior BA first, posterior BA second):

The right increased coupled inhibition between brain areas concerned the connections between BA8 and BA7 and the connection between BA37 and BA47. All these regions have been reported to subserve functions compromised/disturbed in schizophrenic patients, such as the executive control of behavior (BA8: Kubler et al., 2006), the perception of personal space (BA7: Lloyd and Morrison, 2008), the attribution of intentions to others (right BA47: Brunet et al., 2000) and decision making (right BA47: Rogers et al., 1999).

The left decreased coupled facilitation between brain areas concerned the connections between BA7 and BA6, BA9, and BA10, as well as the connection between BA8 and BA37. All these regions have been reported to subserve functions compromised/disturbed in schizophrenic patients, such as planning (bilateral BA7: Fincham et al., 2002), working and long term memory (right BA7: Ranganath et al., 2003) and perception of personal space (right BA7: Lloyd and Morrison, 2008), deductive reasoning (left BA6: Reverberi et al., 2007), prospective memory (left BA10: Okuda et al., 2007), attribution of intentions to others (right BA9: Brunet et al., 2000), true and false memory recognition (left BA37: Slotnick and Schacter, 2004).

Our results suggest that the disturbance of these particular functions in schizophrenia is represented in the brain with two distinct electrophysiological mechanisms: increased coupled inhibition (increased delta connectivity) and decreased coupled facilitation (decreased alpha-1 activity).

In conclusion, the results indicate that across subtypes of acutely ill, productive, first episode schizophrenic patients before start of medication, there is a core set of aberrating functional connectivities that impedes appropriate interaction between the posterior brain areas of preprocessing functions and the anterior brain areas of evaluation and behavior control functions, in agreement with the hypothesized functional dissociation between brain regions in schizophrenia.

### Conflict of interest statement

The authors declare that the research was conducted in the absence of any commercial or financial relationships that could be construed as a potential conflict of interest.
